# Roxborough Park Community Wildfire Evacuation Drill: Data Collection and Model Benchmarking

**DOI:** 10.1007/s10694-023-01371-1

**Published:** 2023-02-08

**Authors:** Steve M. V. Gwynne, Enrico Ronchi, Jonathan Wahlqvist, Arturo Cuesta, Javier Gonzalez Villa, Erica D. Kuligowski, Amanda Kimball, Guillermo Rein, Max Kinateder, Noureddine Benichou, Hui Xie

**Affiliations:** 1 Movement Strategies Ltd, London, UK; 2 Department of Fire Safety Engineering, Lund University, Lund, Sweden; 3 GIDAI Group, University of Cantabria, Santander, Spain; 4 Royal Melbourne Institute of Technology, Melbourne, Australia; 5 Fire Protection Research Foundation, Quincy, USA; 6 Imperial College London, London, UK; 7 National Research Council of Canada, Ottawa, Canada

**Keywords:** Evacuation, WUI, Wildfire, Egress, Fire safety, Drill

## Abstract

Wildfires are increasing in scale, frequency and longevity, and are affecting new locations as environmental conditions change. This paper presents a dataset collected during a community evacuation drill performed in Roxborough Park, Colorado (USA) in 2019. This is a wildland–urban interface community including approximately 900 homes. Data concerning several aspects of community response were collected through observations and surveys: initial population location, pre-evacuation times, route use, and arrival times at the evacuation assembly point. Data were used as inputs to benchmark two evacuation models that adopt different modelling approaches. The WUI-NITY platform and the Evacuation Management System model were applied across a range of scenarios where assumptions regarding pre-evacuation delays and the routes used were varied according to original data collection methods (and interpretation of the data generated). Results are mostly driven by the assumptions adopted for pre-evacuation time inputs. This is expected in communities with a low number of vehicles present on the road and relatively limited traffic congestion. The analysis enabled the sensitivity of the modelling approaches to different datasets to be explored, given the different modelling approaches adopted. The performance of the models were sensitive to the data employed (derived from either observations or self-reporting) and the evacuation phases addressed in them. This indicates the importance of monitoring the impact of including data in a model rather than simply on the data itself, as data affects models in different ways given the modelling methods employed. The dataset is released in open access and is deemed to be useful for future wildfire evacuation modelling calibration and validation efforts.

**Supplementary Information:**

The online version contains supplementary material available at 10.1007/s10694-023-01371-1.

## Introduction

Wildfires are an important safety issue in many parts of the world. Such fires threaten both rural and urban areas. These fires may affect the short-term (life safety, infrastructure and the economy—e.g., the case of the Fort McMurray Fire in Canada) and long-term (environmental conditions, social connectivity, community health, tourism, etc.), potentially even affecting the viability of a community (e.g., Paradise in the USA) [10, 33]. The wildfire issue is likely to get worse in the future, due to climate change and population growth in wildland–urban interface (WUI) locations [21]. The wildfire threat is changing and becoming more serious given several physical and environmental factors: (a) more frequent, larger and intense fires, (b) hotter/drier summers affecting larger areas, (c) more severe thunderstorms and associated lightning strikes potentially starting new fires, and (d) stronger winds encouraging the progress of fire fronts. This also means that the regions exposed to wildfires are expanding beyond those historically affected given changing environmental conditions (e.g., South America, Africa and Northern Europe [12].

The size of the population living in and moving to WUI areas is increasing, as it combines rural and suburban conditions deemed to be attractive. Residential populations are therefore growing near/in the wilderness [5]. This means that there are a larger number of residents (especially those with limited direct experience of wildfire events) living in areas vulnerable to wildfires. In addition, the population of many industrialized countries is ageing. This has an impact on a population's resources and their capacity to respond [2, 17]. Communities are also becoming more diverse making the social and cultural attributes of our communities more complex along with their response [19, 30].

The future expansion of the WUI poses severe challenges to community safety from an evacuation perspective. Large wildfires are associated with severe negative consequences including mass community evacuation, property and livelihood losses, social disruption, damage to infrastructure, as well as evacuee and responder casualties [10]. This has implications for example on the residents of such areas, community planners, emergency managers, the construction industry, and the insurance industry. Such a community might be required to evacuate to a place of relative safety in response to the conditions faced. The time for the community to reach safety is crucial as it determines whether residents avoid worsening (and possibly untenable) conditions [11, 32]. The time for a community to reach a place of safety is an emergent property of its response, the infrastructure available, the information available, and the fire conditions experienced.

It is not possible to extract an accurate estimate of the threat posed by an incident simply by examining any one aspect of a wildfire in isolation.^1^ It is similarly not possible to extrapolate from the performance of one community to another with any confidence, or to a different scenario (e.g., a different location), given that outcomes are extremely sensitive to local conditions. Any assessment requires a coupled approach to provide insights into the vulnerability of certain communities and better inform the preparatory or response actions required. Assessing evacuation performance is key to emergency planning and real-time emergency response. This includes estimating how conditions evolve, how resources are allocated, and quantifying community evacuation given the procedures and routes available.

Previous attempts to map the areas vulnerable to wildfires may become outdated—given the evolving wildfire landscape and associated conditions—affecting community planning and resource allocation. Risk analysis entirely based on historical data is undermined given the speed at which the conditions are evolving leading to very different scenarios being faced [9]. Conclusions drawn from apparently similar previous fires are becoming less relevant—as the underlying factors diverge. The complexity of wildfire events makes it extremely difficult to derive outcomes analytically, and now real-world event-level outcomes (e.g., from historical incidents) are providing fewer directly translatable insights. To understand how these scenarios might evolve now requires (1) a more fundamental understanding of the factors that contribute to the outcome of wildfire evacuations and (2) the development of tools that can assess such events (e.g., quantify them) by taking such factors into account and therefore support regulatory structures that help ensure good practice in this domain. The development of those tools relies on the availability of data. For instance, trigger buffer models need information concerning the time needed by a community to evacuate [16].

Based on these premises, we present data collected from a community evacuation exercise. This data is compiled such that can be employed within evacuation models. Here, two such models (WUI-NITY [31] and evacuation management system, EMS [4] are configured using this data to simulate the original exercise, to establish the capacity of such models to capture the complexity of wildfire evacuation (and explore the underlying dynamics driving these events), and to examine the sensitivity of such models to different interpretations and use of real-world datasets. This should provide insights into the suitability and robustness of such models to community evacuation scenarios and associated engineering applications.

## General Approach: Data Collection and Model Application

The objective of the work presented here is threefold:

- To present data in a format for future wildfire evacuation modelling,
- To demonstrate how models might be used to explore evacuation scenarios,
- To explore and compare the results produced by these models—to determine their stability and applicability to such scenarios.

We gathered data from a community evacuation drill (discussed in Sect. 4). This involved conducting observations and surveys to capture results on key response variables: initial delays, route use and arrival times. The data collected was used to configure the initial conditions within the two models applied (WUI-NITY and EMS)—primarily addressing population size and distribution, initial resident delays, routes available and used, and the target for the evacuating resident population. The original conditions were approximated by simulating nine scenarios based on the different data available (to establish the sensitivity of the model to underlying datasets), or interpreting this data in slightly different ways. This primarily applied to the pre-evacuation times and the use of the available routes. In all scenarios, the original population size involved in evacuation exercise was employed—allowing the results to be directly compared against the evacuation times produced during the exercise.

The following approach was adopted—with data collection explicitly designed to enhance our understanding and to allow the configuration of evacuation models. Initially, we identified a set of factors that might affect the performance of the community evacuation. We developed a data collection plan that focused on collected data on these factors. Data on these factors were collected during the event by staff positioned at key points in the community (e.g. near to the communities and at the assembly point) and through surveying those involved (with surveys completed on the same day). This data was then analysed to quantify the factors previously identified, and also produced in a format that is amenable for model configuration. The data collected was then used to configure the simulation tools employed here. The data was derived from the two sources (direct observation and survey responses) and in some cases produced subtly different indications of performance. This variation was reflected in the model configuration—producing the nine scenarios examined. Each scenario was simulated by the two models allowing comparison—both in their capacity to capture the key evacuation dynamics and their sensitivity to changes in the initial conditions simulated. The modelling approach in use is the so-called “specified calculation” approach [15] in which the user is able to have a detailed description of model inputs, with the goal of performing a comparison of the underlying algorithms in the models.

## Model Descriptions: WUI-NITY and EMS

Based on the objectives of this work, two models (WUI-NITY [25, 31] and EMS [6] have been selected to represent the conditions observed in the drill. The selection of those models was based on their availability and their diverse approaches in modelling the evacuation processes. The intention is to show that different approaches might be used to capture the evacuation process and that useful insights can be gained assuming a suitable understanding of the modelling assumptions made.

### The WUI-NITY Model

WUI-NITY is a simulation platform developed using the game engine Unity 3D. WUI-NITY represents three different modelling layers, namely (1) a fire spread model, (2) a pedestrian response and movement model, and (3) a traffic model. The current implementation of the tool includes macroscopic sub-models for all three modelling layers. In this work, the pedestrian response and movement model and the traffic model have been used. The pedestrian response can be calibrated adopting user-defined curves while the traffic model used in this application is a simple macroscopic traffic model [7]. The traffic model is implemented using time-step intervals. Route choice modelling is performed adopting the open source tool Itinero,^2^ which is able to represent dynamic route choice making use of different overall assumptions (e.g. shortest path or quickest path). The WUI-NITY platform is able to produce a range of outputs, including the number of vehicles at destination or in the road network, the vehicular density, therefore being able to produce evacuation time curves at each destination. Cumulative distributions can be introduced to represent key inputs (e.g. pre-evacuation times, pedestrian walking speeds).

### The EMS Model

The EMS model [6] was developed in.NET Framework 4.6.1. The architecture follows a client–server approach providing an API REST (representational state transfer application program interface) able to integrate data from external services and provide results to other systems. Routing is addressed by the assembly points model (APM) that processes geographical information system (GIS) information and discretizes the evacuation area to generate the locations where pedestrians access the vehicles by considering the population distribution,^3^ the points of reference within the evacuation area (assumed to be close to the road network),^4^ and the random walking distances (from households to assembly points). The shelter points model (SPM) provides a set of available shelters. The location of shelters is slightly modified to well-known points of interest (Pois) close to the road network and directions are indicated using the Open Route Service.^5^ The routing model (RM) uses a local dedicated service to generate the evacuation routes from assembly points to shelters while ensuring the allocation of evacuees to the shelters. EMS simulates movement at the pedestrian and vehicle level (i.e. microscopically), and treats the results stochastically. This means that results are produced as a range of possible outcomes. Two real-time stochastic models based on principles and approaches reported in Cuesta et al. [4] are used by the EMS to simulate mass evacuations (representing pedestrian and vehicular movement). The pedestrian simulation model (PSM) computes the number of individuals entering the vehicular model over time. Each pedestrian is simulated using four random variables: (1) starting location, (2) response time, (3) walking speed (representing movement between residence and vehicle) and (4) vehicle boarding time. The vehicular evacuation model (VEM) simulates, via cellular automata, the current traffic status of the routes and the interactions between vehicles [1, 18] and calculates the number of evacuated vehicles and people arriving to shelters (destinations) over time and the traffic conditions per route (i.e., traffic density and average speed). EMS implements the pre-evacuation times according to log-normal distributions.

## Data Collection: Methodology and Results

Any evacuation model requires data for configuration, calibration and validation [28]. This is essential for the model to be applied to specific scenarios of interest in a credible manner, irrespective of the application type.

**Figure 1:**
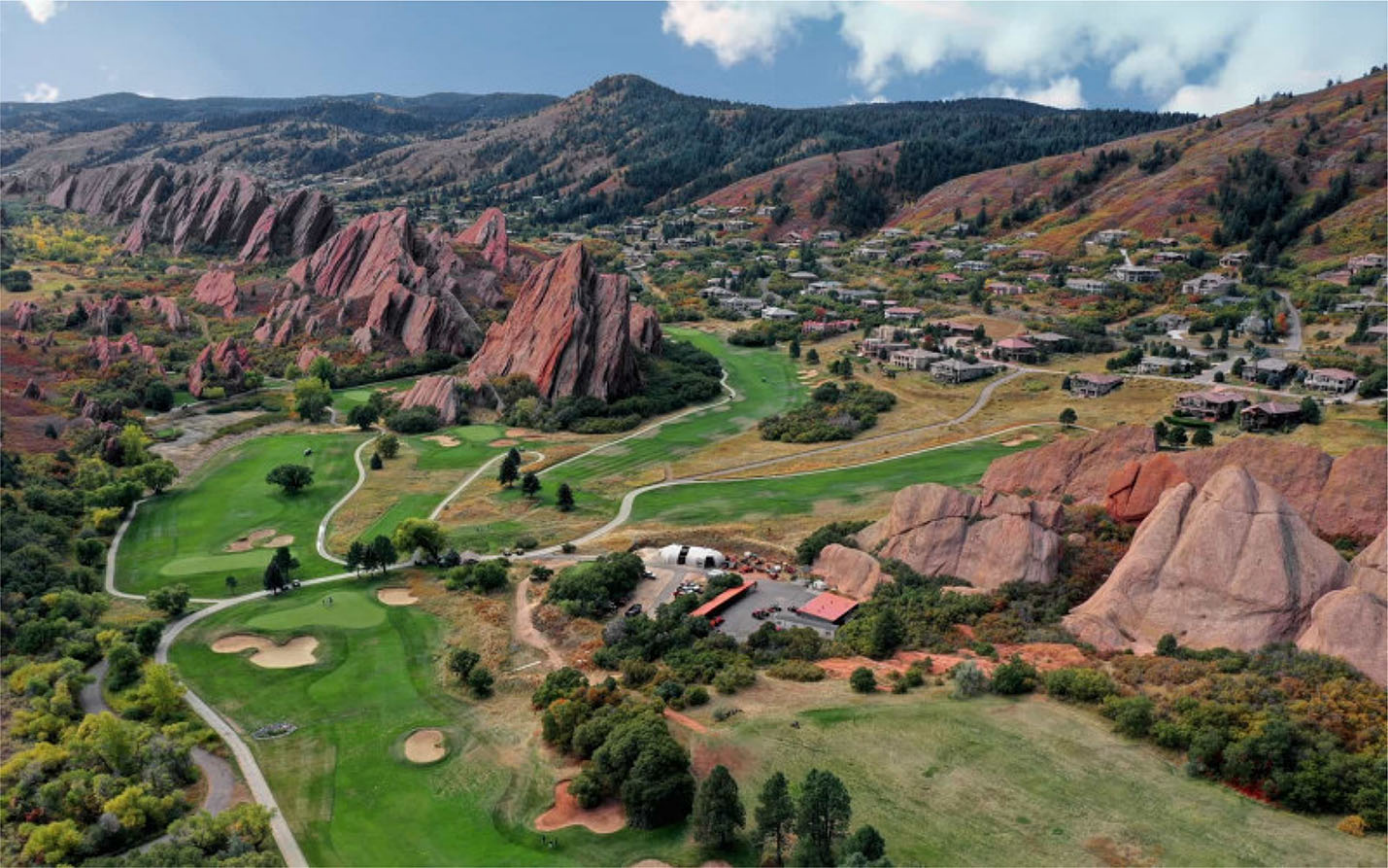
The Roxborough Park community in Colorado, USA. Photo from Perington Miller & Co

A community evacuation exercise was observed in 2019 and data collected on various aspects of the community response: initial population location, pre-evacuation times, route use, and evacuation times (i.e. arrival at the assembly point). Although the intention of the prearranged drill was to enhance the community’s evacuation performance, our data collection efforts were intended to provide insights into the underlying evacuation dynamics and generate a dataset suitable for evacuation modelling, to quantify evacuation performance. As such, the data collection effort was designed to answer practical and research questions. This 2019 exercise involved the Roxborough Park community in Colorado (US).^5^ Roxborough Park is a WUI community that is surrounded by Roxborough State Park on three sides (Roxborough Park Foundation 2007), including approximately 900 homes and covering approximately 8.98 km^2^ (see Figure 1). Roxborough Park was previously exposed to two wildfire events (the 1996 Buffalo Creek Fire and the 2002 Hayman Fire), requiring officials to develop a wildfire protection plan that includes the use of evacuation strategies.

**Figure 2:**
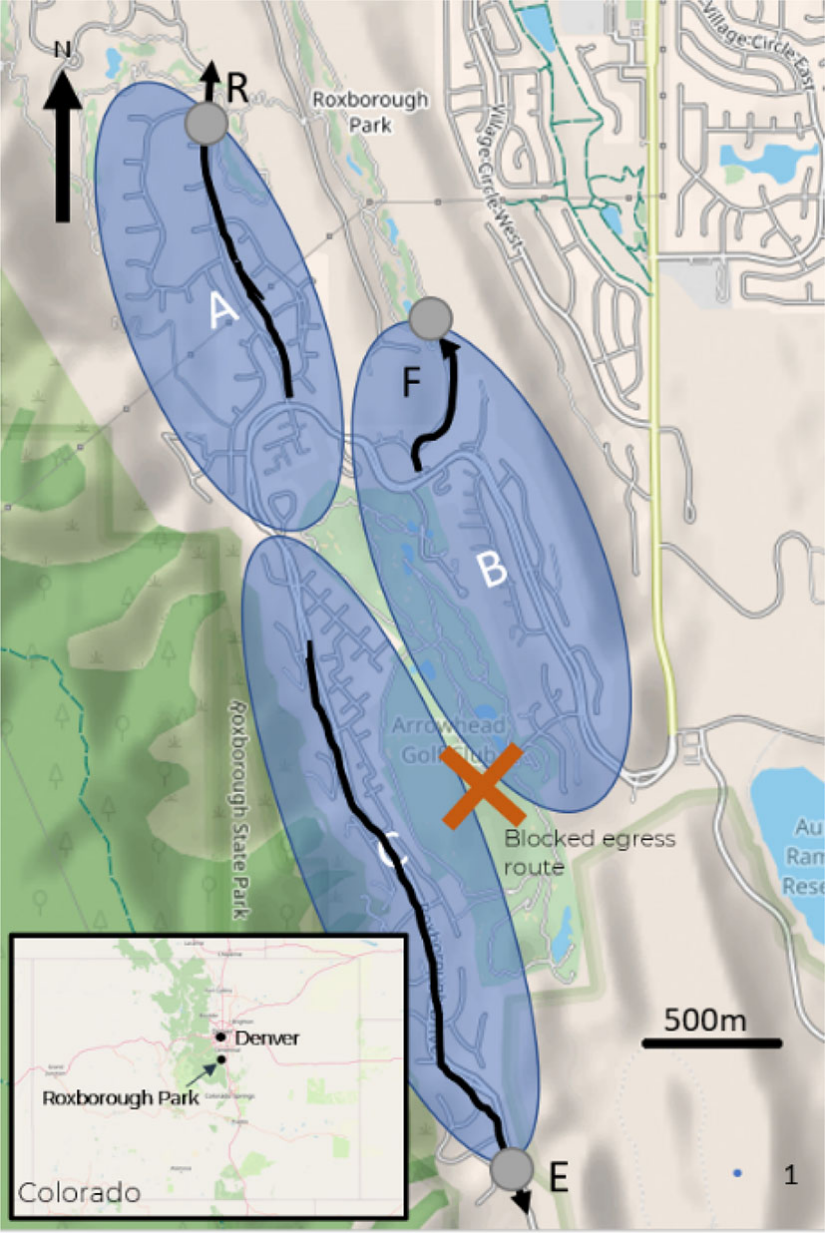
Area involved in the evacuation and incident timeline. *Shaded areas* (A–C) show residential locations. *Arrows* show active egress routes (labelled R, E and F), *orange circles* show approximate location of gates and the inset shows location of Roxborough in relation to Denver. *Map source* OpenStreetMap (Color figure online)

The conditions present at the time of the drill are now described. A full description of the original event can be found here [25]. The community had three primary egress routes (labelled as R, F and E in Figure 2), with an extra route across a golf course (not used during the drill). These three routes were accessed via gates located close to the community housing (identified as circles attached to the community areas labelled A–C in Figure 2). Observations were conducted at these gate locations. The observations at the gate are used as a conservative estimate of pre-evacuation time. The gates were located very close to the communities (shown in Figure 2), and so any overestimate would likely be very small. It should be noted that the drill involved vehicle evacuation and no fire conditions were present or simulated.

The residents had prior knowledge of the drill. A total of 133 households registered to participate with the event taking place on a Saturday morning. It is speculated that another 5–10 households joined the drill during the event without registration (143 in total). Before the drill, registered participants received information on the evacuation routes available. On the day of the drill, an alert was given at 9.00 a.m. to the participants (via text, email, phone call, etc.). After receiving the alert and occasionally engaging in preparatory actions, participants accessed their vehicles and selected one of the evacuation routes available to reach the pre-determined assembly point (at a local fire station). It is expected that the observed delays are optimistic given the prior notice afforded the participants enabling earlier preparedness.

Surveys were conducted to determine where people started, the routes they used, and estimates of the time they left home by car and the time they arrived at the assembly point. Survey results included 69 reported initial delays (pre-evacuation times) and 75 arrival times at the assembly point (overall evacuation times). Observations were made at the three gates (identifying the number of vehicles using each route and arrival times as a proxy for initial delays) and at the assembly point (as an estimate of the overall evacuation time). Observers compiled 107 data points for vehicles at the three gates and 53 arrival times were recorded at the assembly point. Once the evacuation drill was completed, the participants then met with the drill organizers at the assembly point to hand in their completed surveys and participate in a drill debrief. The observations made during the drill were used to configure the simulation tools to provide a benchmark against which model performance might be compared.

**Figure 3:**
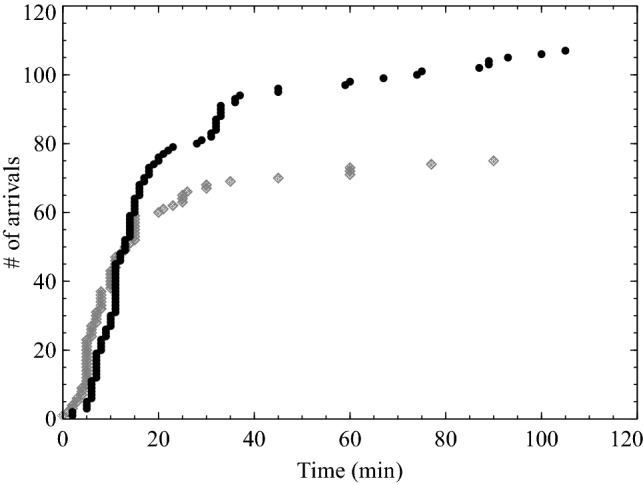
Pre-evacuation times derived from gate observations (in black) and reported in survey (in grey). The self-reported initial delays could also be broken down into the starting locations of the three areas within the community (A–C). Variation is apparent (see also Table 1)

From the surveys and the observed arrival times at the three gates (which given their proximity to the starting locations were used as conservative estimates of the time for people to leave their properties), pre-evacuation delays were estimated to range between 2 and 105 min (see Figure 3). Variations existed in the initial delays—between the observations collected at the gates (which averaged at 23 min, ranging between 2 and 105 min) and the self-reported initial delays (which averaged at 15 min, ranging between 0 and 90 min). This difference is evident in Figure 3 (the black dots represent observed data, the grey dots represent the reported data). Please notice that the number of arrivals is different given the fact that two types of data collection methods were used (i.e. lower number of reported data by answering the survey compared to observations). The higher observed times might have been influenced by the journey from the residences to the gate.

The survey indicated that the population split unevenly between the three available routes (24% used Route F, 45% used Route R, and 31% used Route E). The use of the routes was sensitive to the starting location of the residents. From the survey, 34% started in Area A, 20% in Area B, and 46% in Area C.

**Figure 4:**
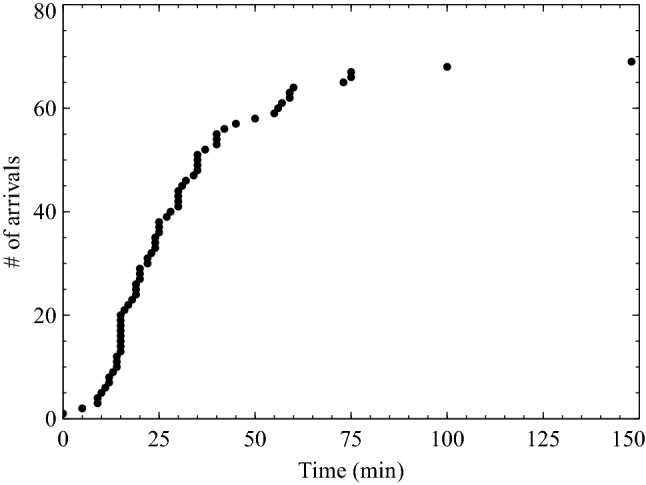
Evacuation times shown as community arrivals over time

Arrival times at the assembly point were generated from survey response estimates and observations. The reported estimates are shown here as they are determined to be more consistent with the assumptions made in the simulations (for instance, people moving directly to the assembly points). Both the survey and observations of arrival times extended to over 135 min.^6^ The reported estimates produced an average evacuation time of 31.1 min, ranging from 0 to 148 min. The arrivals at assembly points are presented in Figure 4.

## Evacuation Simulations

This section introduces the simulation work performed with WUI-NITY and EMS to reproduce the scenario conditions observed in the drill. The scenarios are first presented and then the results of the two models are compared against the data collected. It should be noted that the simulations have been run a posteriori, i.e., the model users were aware of the data available when calibrating the models. This approach has been used since both models are at an initial stage of model development, thus it was deemed useful to reduce the so call *user effect*, i.e., the impact of modeller decisions on results [13]. This allows us to perform a systematic comparison of the predictive capabilities of the models and identify if the order of magnitudes of the results produced are comparable among models and against the data.

### Evacuation Scenarios and Modelling Assumptions

A list of assumptions has been used to configure the models. Population size in terms both of number of people and vehicles on the road was set to resemble the drill conditions. Two key variables have also been modified in different scenarios in order to investigate their impact on results. These are the pre-evacuation time distributions used and the evacuee route selection.

#### Population Size

The number of vehicles in the simulation is assumed to match the number of households that were present during the drill (this was assumed equal to 143 households). This implies that each household evacuated using only one vehicle. It should be noted that since the evacuation drill included only private vehicles (e.g., cars), no public transportation means (e.g., buses) are considered in the scenarios.

#### Pre-evacuation Time Distributions

Three different sets of assumptions were used for the pre-evacuation time distributions. These were based on the data collection approaches used in the evacuation drill and the level of detail captured. The first assumption (coded as PEvacES) uses data based on observations by each gate (see Table 1). The second assumption employs a distribution generated from the self-reported data derived from completed surveys (PEvacCSR). The third assumption employs a combined cumulative curve from gate observations combined across zones (PEvacCES).

**Table 1:** Initial Delay Times by Community Area

Area	Reported pre-evacuation times (min)
Overall	15.0 [0–90]
A	14.4 [0–90]
B	16.7 [0–60]
C	14.5 [1–90]

#### Destination Usage

Three different assumptions have been used for destination usage, namely (1) each group (A, B, or C) will go to destinations R, F, or E according to survey route use from the drill (coded as DestDrill), (2) vehicles use the closest destination as defined by the model (coded as DestClose), (3) vehicles use the destination associated with the fastest route as defined by the model (coded as DestFast).

The combinations of these assumptions result in a total of nine scenarios. Table 2 reports the scenario names along with the assumptions in use.

**Table 2:** Scenarios Investigated Considering Different Assumptions for Pre-evacuation Time Distributions and Destination Usage

Scenario name	Pre-evacuation time	Destination usage
1_PEvacES_DestDrill	PEvacES	DestDrill
2_PEvacCSR_DestDrill	PEvacCSR	DestDrill
3_PEvacCES_DestDrill	PEvacCES	DestDrill
4_PEvacES_DestClose	PEvacES	DestClose
5_PEvacCSR_DestClose	PEvacCSR	DestClose
6_PEvacCES_DestClose	PEvacCES	DestClose
7_PEvacES_DestFast	PEvacES	DestFast
8_PEvacCSR_DestFast	PEvacCSR	DestFast
9_PEvacCES_DestFast	PEvacCES	DestFast

#### Outputs

A set of outputs are produced by the models. These include (1) the average evacuation time per scenario for different percentages (95%, 99%, 100%) of evacuated populations (these results are presented in “Online Appendix 2”), and (2) the aggregated evacuation time curves, i.e., number of vehicles evacuated over time. Therefore, all three indicators (95%, 99%, 100%) are directly compared and the characteristics of the entire evacuation curves are compared using functional analysis. As both models adopt pseudo-random sampling from distributions, a criterion was defined to ensure the convergence of the multiple runs is met [24, 27]. In this case study, we assumed that convergence is met once the average total evacuation time did not variate for more than 2% over at least 10 consecutive runs. This was estimated to be a reasonable value given the overall uncertainty in the models and data in use.

Once the average evacuation times and aggregated evacuation time curves are then produced with both models, those are plotted against the drill data to allow visual qualitative comparison of the curves obtained. In addition, functional analysis [20] is employed to perform a quantitative comparison of the results. This method makes use of three different operators that can be applied to the evacuation time curves, namely the (1) Euclidean relative distance (ERD), the (2) Euclidean projection coefficient (EPC) and (3) the secant cosine (SC). ERC indicates the average difference between curves. EPC is used to represent the projection of a vector onto another, and it helps understanding the best possible agreement between the curves. EPC represents a factor which when multiplied by each model data point reduces the distance between the experimental data and the model. SC indicates the similarity between the shapes of the curves by assessing the gradients they produce. For instance, two curves with identical shapes (even if translated of a given distance) would have an SC equal to 1. A more detailed discussion of these concepts can be found in Ronchi et al. [24]. These produce three dimensionless metrics: ERD (with a value of 0 indicating an exact match of the experimental and modelling results curve in magnitude), EPC (with a value of 1 indicating an exact match, i.e. the difference between the model and experimental data are as small as possible), and SC (with a value of 1 indicating an exact match between experimental data and model results). These metrics are used to indicate similarity of the simulated curves and the data collected.

### WUI-NITY Simulation Results

**Figure 5:**
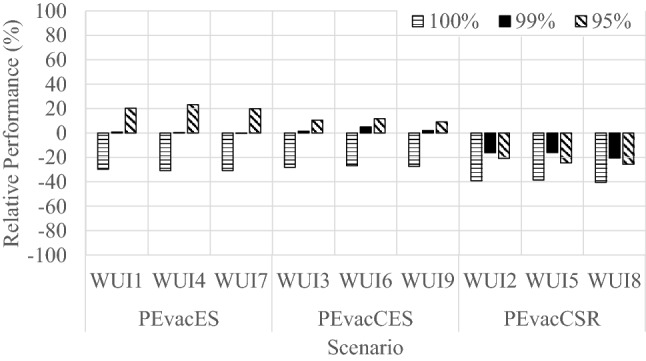
Relative evacuation performance generated by WUI-NITY in relation to the drill performance (SR_DRILL) (95%, 99% and 100%). WUIX indicates the model in use (WUI = WUI-NITY) and the scenario under consideration (e.g., 1, 2, etc.)

The average results produced by WUI-NITY relative to the original drill data are presented in Figure 5. It is apparent that the WUI-NITY model typically underestimated the original evacuation times, with some notable exceptions. Figure 5 includes the 95%, 99% and 100% of aggregated average evacuation times. The evacuation times generated by WUI-NITY are reported in “Online Appendix 2”.

Regardless of the scenarios under consideration, WUI-NITY results show an overall better agreement to the 99th percentile of the drill evacuation time than for the 95th and 100th percentiles (with differences ranging between 9 and 26% for 95th percentile, 1% and 20% for 99th percentile, and 27% and 40% for the 100th percentile, see Figure 5 and “Online Appendix 2”). This is likely due to the modelling approach employed where inputs are represented by a cumulative distribution within WUI-NITY. The WUI-NITY model is also a macroscopic model. As such the interactions between evacuating residents and vehicles are not represented at the individual level. Therefore, the consequences of individual interactions are captured at the aggregate level—implying that the model is inherently less sensitive to such factors.

Scenario WUI3 provides results directly comparable to the drill data for the 99% evacuation time percentile (with a difference below 1%). This scenario makes use of the inputs that reflect drill conditions (route use is based on that observed in the drill, and pre-evacuation times were derived from compiling arrival times observed across all gates).

It is apparent that the results of Scenarios WUI2, WUI5 and WUI8 (all PEvacCSR scenarios—employing self-reported pre-evacuation times) consistently yield *lower* evacuation times—that deviate most from the drill results. This is to be expected, given that these scenarios employ pre-evacuation time data from the self-reported data, which have lower frequencies of occurrence towards the tail of the distribution—making lower pre-evacuation times more likely to be assigned to the simulated residents. They also might exclude movement between individual properties and the local gate—where the vehicle effectively enters the widely traffic system.

The remaining scenarios (WUI1, WUI4, WUI6, WUI7 and WUI9) that are not entirely reliant on self-reported pre-evacuation times produce results that vary. Nevertheless, they approximate the 99% reasonably well (falling within 5% of the time recorded during the drill). Generally, WUI-NITY approximates the evacuation times reasonably for those scenarios making use of inputs that most closely resemble the drill conditions when compared against the 99% of the evacuation times. Comparison is less satisfactory when compared against the 100% evacuation time, although the final evacuation time during the original drill was very sensitive to the arrival time of a single vehicle that was a significant outlier.

**Figure 6:**
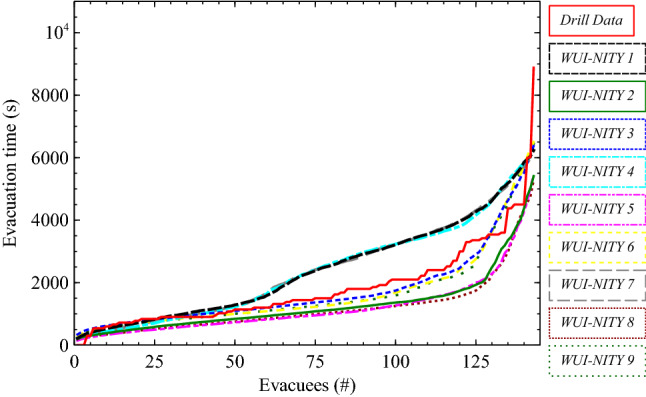
Aggregated evacuation time curves reported during the drills compared to the results of the WUI-NITY evacuation simulation platform

The evacuation curves for each of the WUI-NITY scenarios examined were compared against the drill curve using functional analysis (see Figure 6). It is apparent that Scenarios WUI2, WUI5 and WUI8 consistently under-predicted the drill evacuation times across the event. Scenarios WUI3, WUI6 and WUI9 more closely approximate the evacuation time curves, while Scenarios WUI1, WUI4 and WUI7 provide overall more conservative results (considering the curve up to 99%). The results seem to indicate that—in this case study—results are mostly driven by the assumptions adopted for pre-evacuation time inputs. This is reasonable, given the relatively low number of vehicles present on the road which makes results less dependent on route choice and traffic congestions.

The results obtained from the graphical inspection of aggregated evacuation time curves are confirmed by the quantitative comparison with drill data performed with functional analysis (see Table 3).^7^ The larger values for ERC can be observed for Scenarios WUI2, WUI5 and WUI8 (ERD = 0.42, 0.48 and 0.52 respectively), while lower ERC values are reported for Scenarios WUI3, WUI6 and WUI9 (ERD = 0.16, 0.18 and 0.19 respectively). As ERC is an indicator of the overall agreement between the curves, these results are expected—and agree with our previous findings regarding the most representative scenarios. According to the underprediction of evacuation times, EPC values are higher in Scenarios WUI2, 5 and WUI8 (EPC = 1.17, 1.20 and 1.21 respectively). The closest value to 1 (indicating better agreement with the drill curve) for EPC is observed in Scenario WUI3 (EPC = 1.01), followed by Scenario WUI6 (1.02) and Scenario WUI9 (1.03).

**Table 3:** Quantitative Comparison Between Drill Data and WUI-NITY Simulation Results

Scenario number	ERC	EPC	SC
WUI1	0.29	0.87	0.61
WUI2	0.42	1.17	0.75
WUI3	0.16	1.01	0.67
WUI4	0.29	0.87	0.54
WUI5	0.48	1.20	0.79
WUI6	0.18	1.02	0.65
WUI7	0.29	0.87	0.60
WUI8	0.52	1.21	0.69
WUI9	0.19	1.03	0.60

The agreement between drill data and aggregated evacuation time curves is also sensitive to the assumed inputs for pre-evacuation time. This results in the scenarios making use of the estimated pre-evacuation times based on observed arrival times per gate being associated with higher differences in curve shapes with the drill data (SC = 0.61, 0.54 and 0.60), followed by the observed pre-evacuation times based on observed arrival times across gates (SC = 0.67, 0.65, 0.60), with the highest SC scores recorded for the self-reported pre-evacuation times (SC = 0.75, 0.79, 0.69). The best agreement in terms of curve shapes (drill data vs. scenarios) is found for Scenario WUI5 (SC = 0.79).

It is apparent that Scenario WUI3 has the best ERC and EPC score, and the fourth best SC score (the best outside of scenarios with reported pre-evacuation times). This represents the best overall performance, consistent with the earlier findings.

### EMS Model Simulation Results

**Figure 7:**
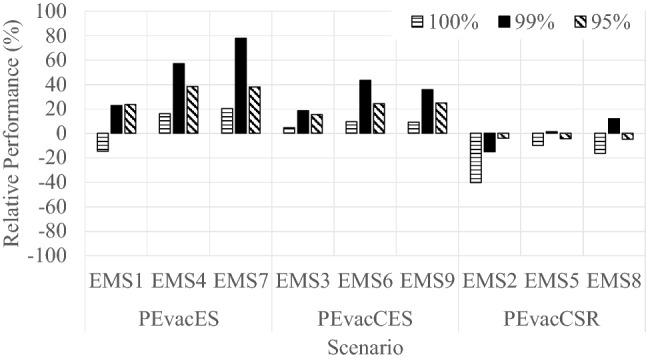
Relative evacuation performance generated by EMS in relation to the drill performance (SR_DRILL) (95%, 99% and 100%)

Figure 7 shows the relative errors produced by the EMS for 95%, 99% and 100% of aggregated average evacuation times compared with the drill data. Typically, EMS *overestimates* the evacuation time, with a few notable exceptions. For clarity, the scenarios in Figure 7 are grouped according to pre-evacuation inputs (observations per gate, self-reported, observations compiled across all gates). Differences ranged between 4 and 38% for the 95th percentile, 1% and 78% for the 99th percentile, and 5% and 40% for the 100th percentile. The EMS overpredicts the evacuation times (95%, 99%, and 100%) for scenarios with pre-evacuation data derived from observations at the gates which included both the initial delay and the travel times from house to vehicle and from vehicle to gate (PEvacES and PEvacCES). Note that these times were assumed in the model to represent the time when people left their properties and that the model represented the other two additional time components explicitly (i.e., travel time to the vehicles and boarding vehicle times), along with movement from the specific premises to the community gate.

As shown in Figure 7 the model produced the largest relative differences (for the 95th 99th and 100th percentiles of evacuation) for Scenario EMS1 (0.23, 0.23 and − 0.15), Scenario EMS4 (0.38, 0.57 and 0.16), and Scenario EMS7 (0.38, 0.78 and 0.20) in which pre-evacuation times were implemented according to observations by each gate (PEvacES). In addition, the EMS implements the pre-evacuation times according to log-normal distributions while the gate arrival observations separately (route R, F, and E) did not fit against this distribution. This may also contribute to the relatively large differences.

In Scenarios EMS3, EMS6 and EMS9 the pre-evacuation times were represented by observations compiled across the gates. This produced lower relative differences (for the 95th, 99th and 100th percentiles of evacuation) than those outlined above (Scenario EMS3 produced differences of 0.15, 0.18 and 0.04; Scenario EMS6 produced differences of 0.24, 0.43 and 0.09; Scenario EMS9 produced differences of 0.24, 0.35 and 0.09). This may have been due both to reduced specificity of the results (compiling across gates), leading to the PEvacCES data sample fitting a log-normal distribution thus reducing the frequency and impact of extreme values when generating pseudorandom inputs.

The best agreement between the EMS and the drill data was found for the PEvacCSR scenarios, where pre-evacuation times were based on self-reported estimates—more closely reflecting the elements included within the pre-evacuation phase within EMS (i.e., the time from the warning to leaving property). The model underpredicted the drill data in Scenario EMS2 (produced differences of − 0.04, − 0.15 and − 0.40), Scenario EMS5 (produced differences of − 0.04, 0.01 and − 0.09) and Scenario EMS8 (produced differences of − 0.04, 0.12 and − 0.16). As mentioned, a possible explanation for these underestimations may be the self-reported data used with lower frequencies of occurrence towards the tail of the distribution—making lower pre-evacuation times more likely to be assigned to the simulated residents. However, although under-predictions, they were the best estimates of the 100th percentile evacuation performance.

**Figure 8:**
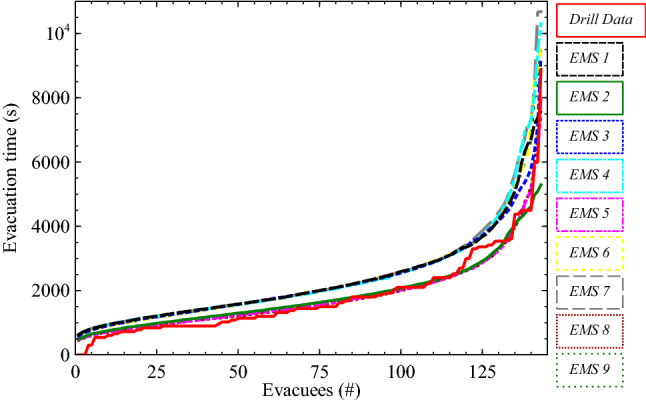
Aggregated evacuation time curves reported during the drills compared to the results of the EMS

The comparison of the aggregated evacuation time curves is shown in Figure 8. Results in Table 4 show a fair agreement for ERC values in scenarios (EMS1, EMS2, EMS3, EMS5, EMS6, EMS8 and EMS9). The EPC values for all scenarios were higher than 0.85 and lower than 1.03 whereas SC values (> 0.80) were produced for scenarios (EMS3–EMS9) suggesting reasonable predictions of the evacuation curves generated by the EMS.

**Table 4:** Quantitative Comparison Between Drill Data and EMS Simulation Results

Scenario number	ERC	EPC	SC
EMS1	0.23	0.90	0.64
EMS2	0.18	1.02	0.72
EMS3	0.20	0.91	0.94
EMS4	0.26	0.87	0.85
EMS5	0.09	1.01	0.92
EMS6	0.24	0.88	0.85
EMS7	0.28	0.86	0.83
EMS8	0.11	1.01	0.87
EMS9	0.23	0.89	0.88

The comparison between ERC, EPC and SC results is more complex for the EMS results; however, it is apparent that generally, consistent with the findings above, the best results were generated for those scenarios employing the self-reported estimates of pre-evacuation (Scenarios EMS2, EMS5 and EMS8).

## Discussion

This paper presents a new dataset concerning community evacuation. This is deemed an original contribution to the research literature, as such type of datasets for wildfire evacuation scenarios are rarely available (in contrast with data from building evacuation drills which are much more common and reported in fire engineering handbooks [8]. This work demonstrated that such data can be useful not only for enhancing preparedness of a given community, but also to increase the understanding of evacuation model predictive capabilities. The field of wildfire evacuation modelling is relatively new [23], thus it is recommended that such data are for now used performing mostly a posteriori simulations, with the scope of evaluating the overall agreement of the model results with known evacuation processes. The process of input calibration also allows for testing the sensitivity of models to different types of inputs, and in turn perform an evaluation of the field of application of given models in relation to their modelling assumptions.

The evacuation dataset was applied to the WUI-NITY and EMS models that adopt different modelling approaches: the former employs a macroscopic approach while the latter applies a microscopic approach applied stochastically. Results of both models show that the key variable influencing evacuation, time in this particular case, is the response of the population; i.e. the pre-evacuation delay modelled. This is not surprising given the fact that the WUI community under consideration is in a rural area with a relatively low number of households—this means that the evacuating traffic demand does not overload route capacity, with conditions being more sensitive to the departure profile than demand. In such scenarios, the response distribution instead has a dominant impact since congestion is less apparent and outliers’ evacuees may significantly influence overall evacuation time. In this context, route selection also had a relatively small impact on results. This was also expected given the fact that the network capacity has not been reached in the given scenarios.

**Figure 9:**
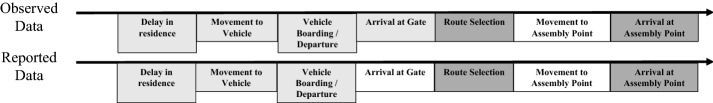
Performance elements included in the observed and reported data

This work represented an unusual, albeit fortuitous, opportunity to explore the impact of using different datasets from the same incident on the use of two different modelling approaches. Observations were made during the original incident—covering movement up to the arrival at a set of gates located near to each sub-community. In addition, those arriving at the assembly point completed a survey where they estimated the time it took them to leave their properties. Although similar, the former data includes a journey from the residence to the gate of the community, while the latter only extends up to the point residents leave their home (see Figure 9).

WUI-NITY does not represent movement to vehicles or vehicle boarding explicitly and is seen to typically underestimate the total evacuation times in this case. Therefore, the movement stages represented in the observed arrival at the gates is a better fit given that it includes elements not explicitly captured in the WUI-NITY model. This is borne out by the simulated evacuation times that employed the CES pre-evacuation times (compiled across the gate observations) producing the best estimates of the drill evacuation.

EMS simulates movement at a more granular level—it is able to explicitly represent local movement that reflects the delay inside the property, movement to the vehicle, vehicle boarding and then vehicle movement. Conceptually, it appears that EMS is then better suited to the self-reported data—which excludes movement to the edge of the community. EMS represents pre-evacuation performance using a log-normal distribution. This provides an additional difference with observations at the gate, given that this distribution deviates somewhat from a strict log-normal shape. These results are borne out in the comparisons made above.

Bearing in mind the differences in modelling approaches adopted by the two models in use, different models may be used in some cases side-by-side for comparison. This could possibly provide a better understanding of the evacuation process and in turn allow for more accurate predictions. A multi-model approach has indeed been already recommended in the pedestrian evacuation domain [22] with the scope of making an optimal use of the strengths of each model.

It should also be noted that, given the methodology employed for data collection, results should be analysed considering that uncertainty is also present in outliers reported in the drill data themselves. There was an outlier in the original results, with the last vehicle arriving 48 min (nearly 3000 s) after the previous vehicle—twice as long as any of the preceding gaps between arriving vehicles. Looking at the results of the WUI-NITY model, it is therefore not surprising that a better agreement is observed excluding this outlier arrival—comparing between the 99th percentile results. Although this outlier was a real data point, WUI-NITY currently makes use of a macroscopic approach which might not be expected to capture such outliers as well as a microscopic approach. EMS was shown to be more sensitive to the double representation of parts of the pre-evacuation phase, while being better able to address the outlier challenge, given the more granular, stochastic approach. However, this led to some instances in which over-predictions of evacuation times were observed. This is contrast with the WUI-NITY results where the closest approximation was obtained with the pre-evacuation assumption of a combined cumulative curve from estimated data.

The analysis reported here demonstrates that the assumptions adopted by the models make them more or less sensitive to different inputs and the way evacuation datasets are incorporated into model inputs. This implies that the process of configuration of a tool requires dedicated efforts, to consider the most appropriate inputs in relation to the assumptions adopted. The user might need to use slightly different datasets to produce equivalent scenario conditions given the use of different models. In other words, data might have different implications on the outcome produced, i.e., leading to more or less conservativism and being sensitive to certain inputs to a larger or smaller extent. Once a user is aware of those limitations, they may be counter-balanced through the use of safety factors or the adoption of a more conservative use of data.

The results produced are encouraging—in that although the datasets that produced the best estimates are different, the differences reflect differences in the modelling logic, functionality or level of granularity. In addition, the best results produced in each of the models occur when credible assumptions of the original drill conditions are made. When these are made, the model estimates (for the 99th percentile for WUI-NITY and the 100th percentile for EMS) are within 5% of the drill time. This is considered encouraging—given that the sensitivity of each model to the datasets used (performance elements reflected in the data, the distribution type, and existence of outliers within the data) is understood and reflected in data selection and model configuration.

It is desirable that the presented dataset represents a starting point—promoting more data collection efforts of this kind. Despite several efforts to collect data, those are generally obtained by actual events through surveys [3, 29, 30, 34], GPS data [35, 36] or social media data [14]. In contrast, evacuation data from community drills allow higher control in the variables to be observed. It is therefore desirable that such type of data collections is facilitated by wildfire safety programmes (e.g., FireWise in the US or FireSmart in Canada) as they may have a positive impact on the predictive capabilities of wildfire evacuation models.

## Conclusion

A dataset obtained performing observations and a survey during a community evacuation exercise conducted in Roxborough Park (Colorado, USA) is presented and data are published in open access. The dataset has been used to calibrate two models, WUI-NITY and EMS. The use of such data for wildfire evacuation modelling has been demonstrated by varying key input configurations on route use and pre-evacuation. Results show that pre-evacuation is the dominant factor in rural wildfire evacuation scenarios with a small number of households.

These results imply that the impact of data on a model performance is sensitive to the methods used within the model—and how these methods apply to different stages of the evacuation process. It is not sufficient just to configure different models using the same dataset to ensure that they are representing the same conditions. It is necessary to understand the combination of model and data, requiring detailed knowledge of both—to fully appreciate the implications of using the data and the extent of calibration necessary.

## Supplementary Information

Below is the link to the electronic supplementary material.

Supplementary file1 (PDF 354 kb)

## Footnotes

1 The same can be said of all fire scenarios—and all emergency scenarios.

2 https://www.itinero.tech/.

3 The default population distribution is taken from WorldPop (https://www.worldpop.org/), a project data which uses both integrating census data and aerial imagery via satellite in which people counts and density are provided with a resolution of 100 m^2^.

4 OpenRouteService (ORS) (https://openrouteservice.org/).

5 It should be noted that any results presented here beyond the original drill do not reflect the current preparedness or vulnerability of Roxborough and are presented here purely to demonstrate the complexity of wildfire evacuation—and the use of a model as a proxy for the complexity.

6 This included by the initial delays and the movement to the assembly point.

7 Reference should be made to Sect. 5.1.4 for definitions of these terms.
