# Supplementary material for: Roxborough Park Community Wildfire Evacuation Drill: Data Collection and Model Benchmarking
Source: Fire Technol. 2023 Feb 8;59(2):879–901. doi: 10.1007/s10694-023-01371-1 (PMC9977886; doi:10.1007/s10694-023-01371-1)
Supplement: Supplementary file 1 — Supplementary file1 (PDF 354 kb) [file 10694_2023_1371_MOESM1_ESM.pdf]

Appendix A – Roxborough Raw Data

Several sets of raw data are presented below. There are occasional gaps in the data. This reflects omissions in the returned surveys.

Gate Arrival Observations – Estimating Pre-Evacuation Times

Pre-Evacuation Time (min)
2
2
5
5
5
6
6
6
6
6
6
7
7
7
7
7
7
7
7
8
8
8
8
9
9
9
10
10
10
10
11
11
11
11
11
11
11
11
11
11
11
11
11
11
11
12
12
12
13
13
13
13
14
14
14
14
14
14
14
15
15
15
15
15
16
16
16
16
17
17
18
18
18
19
20
20
21
22
23
28
29
31
31
32
32
32
32
33
33
33
33
36
36
37
45
45
59
60
67
74
75
87
89
89
93
100
105

Self-Reported estimates of pre-evacuation

Pre-Evacuation Time (min)
0
1
2
2
3
3
4
4
4
5
5
5
5
5
5
5
5
5
5
5
5
5
5
6
6
6
6
7
7
7
7
8
8
8
8
8
8
10

10
10
10
10
10
11
11
11
11
12
13
13
14
15
15
15
15
15
15
15
15
20
21
23
25
25
25
26
30
30
35
45
60
60
60
77
90

Reported Pre-Evacuation by Start Zone

A	5
A	25
A	60
A	10
A	5
A	15
A	30
A	5
A	60
A	8
A	7
A	2
A	30
A	15
A	15
A	11
A	10
A	5
A	5
A	8
A	6
A	0
A	4
A	13
A	6
B	15
B	5
B	7
B	5
B	77
B	6
B	15
B	14
B	15
B	5
B	11
B	6
B	60
B	8
B	2
C	13
C	5
C	5
C	5
C	
C	10
C	8
C	1
C	15
C	21
C	5
C	3
C	35
C	25
C	45
C	11
C	4
C	10
C	8
C	10
C	15
C	90
C	23
C	7
C	25
C	7
C	5
C	5
C	11
C	26
C	10
C	4
C	3
C	8
	20
	12

Route Use by Start Zone

R	A
R	A
	A
R	A
R	A
R	A
R	A
R	A
F	A
R	A
R	A
R	A
R	A
R	A
R	A
F	A
R	A
F	A
R	A
E	A
R	A
R	A
R	A
R	A
R	A
F	B
F	B
F	B
F	B
E	B
F	B
F	B
F	B
F	B
E	B
F	B
F	B
F	B
F	B
F	B
R	C
F	C
R	C
R	C
E	C
R	C
R	C
E	C
E	C
E	C

E	C
E	C
R	C
E	C
R	C
R	C
R	C
E	C
E	C
R	C
E	C
R	C
F	C
E	C
E	C
E	C
E	C
E	C
E	C
R	C
E	C
E	C
E	C
R	C
R	
E	

Reported Evacuation Times (mins)

0
5
9
9
10
11
12
12
13
14
14
14

15
15
15
15
15
15
15
15
16
17
18
19
19
19
20
20
20
22
22
23
24
24
24
25
25
25
27
28
30
30
30
30
31
32
34
35
35
35
35
37
40
40
40

42
45
50
55
56
57
59
59
60
73
75
75
100
148

Appendix B – Results of WUI-NITY and EMS simulations

	% of evacuees	DRILL	WUI1	WUI2	WUI3	WUI4	WUI5	WUI6	WUI7	WUI8	WUI9
**Evac. time (s)**	100%	8880	6234	5410	6382	6142	5466	6500	6151	5274	6440
	95%	4380	5266	3470	4842	5399	3310	4891	5246	3258	4778
	99%	6000	6047	5027	6086	6020	5039	6301	5982	4769	6120
	**% of evacuees**	**DRILL**	**EMS1**	**EMS2**	**EMS3**	**EMS4**	**EMS5**	**EMS6**	**EMS7**	**EMS8**	**EMS9**
**Evac. time (s)**	100%	8880	7580	5290	9280	10310	8000	9730	10680	7420	9680
	95%	4380	5410	4200	5050	6060	4180	5440	6040	4170	5470
	99%	6000	7370	5090	7110	9420	6080	8600	10670	6720	8150

	% of evacuees	WUI1	WUI2	WUI3	WUI4	WUI5	WUI6	WUI7	WUI8	WUI9
**Differences (%)**	100%	-30	-39	-28	-31	-38	-27	-31	-41	-27
	95%	1	-16	1	0	-16	5	0	-21	2
	99%	20	-21	11	23	-24	12	20	-26	9
	**% of evacuees**	**EMS1**	**EMS2**	**EMS3**	**EMS4**	**EMS5**	**EMS6**	**EMS7**	**EMS8**	**EMS9**
**Differences (%)**	100%	-15	-40	5	16	-10	10	20	-16	9
	95%	23	-15	19	57	1	43	78	12	36
	99%	24	-4	15	38	-5	24	38	-5	25

WUI indicates WUI-NITY and EMS indicates the Evacuation Management System. The number indicates the scenario under consideration.
